# Field trials of GM trees in the USA: activity and regulatory developments

**DOI:** 10.1186/1753-6561-5-S7-O61

**Published:** 2011-09-13

**Authors:** Steven H Strauss, Venkatesh Viswanath

**Affiliations:** 1 Department of Forest Ecosystems and Society, Oregon State University, 321 Richardson Hall, Corvallis, OR 97331, USA

## 

Field trials are extremely important for all facets of research, breeding, and biotechnology. Tree physiology is distinctively different in greenhouses or growth chambers vs. field environments. Thus, results from non-field environments can be very misleading with respect to identification of promising biotechnologies and elite varieties. Large regulatory impediments to conduct of field tests of genetically modified (GM) trees will therefore tend to stifle scientific and technological development. This appears to be the case for genetically modified trees, where regulatory burdens to conducting field tests have grown in stringency in the USA and elsewhere in recent years [1,2]. Here, we present the record of recent field trials and some new regulatory developments in the USA.

## Overview of field trials in the USA

**Figure 1:**
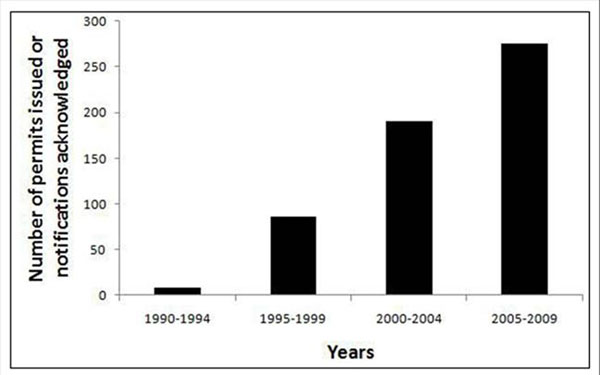
Total number of field trials with transgenic trees in the USA between 1990 and 2009.

Information Systems for Biotechnology (ISB) [3] maintains an easily used database of GM crop data in the United States. There have been nearly 600 field trials (including both “acknowledgments” and “permits”) conducted since 1989, with a five-fold increase occurring in 2000-2009 compared with 1990-1999 (Figure 1).

**Figure 2:**
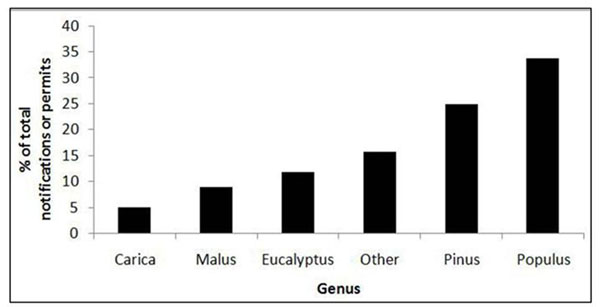
Major genera of transgenic trees in field trials in the USA, 1990 to present.

Fifteen different genera have been tested, including *Populus*, *Pinus*, *Eucalyptus*, and *Malus*, with the first two accounting for approximately 60% of all field trials (Figure 2). Marker genes and tolerance to biotic stresses were the two most common research objectives. Private companies carried out 60% of field trials, and their activity has grown dominant in recent years.

Currently, there are more than sixty field trials covering eleven different genera, with *Populus* accounting for 35% of all field trials. *Eucalyptus*, *Liquidambar*, and *Malus* together account for another 37% of trials. Other genera being tested include *Castanea* (American chestnut), *Pinus*, *Ulmus* (American elm), *Prunus*, *Musa*, *Citrus*, and *Juglans*. Forty field trials are being conducted by private companies, with ArborGen alone accounting for 36 of the 40 active field trials. Cornell University, North Carolina State University, Oregon State University, Purdue University, the United States Department of Agriculture (ARS) and the University of California, Davis are among the public institutions currently conducting field trials. One of ArborGen’s field trials with cold tolerant *Eucalyptus* covers 197.2 acres. This is the largest current field trial in terms of acres and is being conducted in six different states. In terms of research objectives of current trials, marker genes dominate in terms of frequency of trials, with modification of wood quality the next most common objective.

## Regulation of field trials in the USA

In the USA, transgenic trees are regulated in the same manner as agricultural crops [4], using regulatory laws created prior to the development of transgenic biotechnology. The three agencies involved are the United States Department of Agriculture (USDA), which considers agricultural safety and economics; the Food and Drug Administration (FDA), which considers human and animal feed safety; and the Environmental Protection Agency (EPA), which considers pesticidal properties of transgenic organisms [5]. Regulations in the United States are perceived to be less stringent than those in Europe and Asia, however, a recent survey in the USA found that forest scientists—including breeders, biotechnologists, and ecologists—see regulations as major obstacles to field research and commercial development of GM trees [6].

## Recent regulatory developments

A company known as Okanagan Specialty Fruits has taken the lead in the production of transgenic tree varieties produced by the insertion of cisgenes and intragenes [7]. Non-browning versions of established apple varieties were developed by the company by the silencing of the polyphenol oxidase gene. These apples have been named Arctic^TM^ apples because of the color of their skin. The company has petitioned the Animal and Plant Health Inspection Service (APHIS) for deregulation of the product in the USA [8].

Cold tolerant eucalyptus varieties were developed by ArborGen [9]. The company believes that these varieties would permit the planting of highly productive eucalypt hybrids for bioenergy and pup north of Florida in the USA. In May 2010, the USDA authorized a large scale flowering field trial of these trees, thus permitting the planting of nearly 260,000 trees over ~300 acres in 7 states [10]. However, several environmental organizations filed a lawsuit against APHIS because they believed that APHIS performed an inadequate environmental analysis, thus violating the US National Environmental Policy Act (NEPA).

A new two year pilot project has recently been announced by APHIS [11]. Under the provisions of the new project—whose aim is to speed up the review process before deregulation of biotech crops—environmental assessments of transgenic crops will be conducted by companies themselves, or by contractors authorized by the USDA. Although USDA would still need to review and approve the analyses, critics of the new policy believe that it might lead to less stringent environmental analyses.
